# Hypocretin in the nucleus accumbens shell modulates social approach in female but not male California mice

**DOI:** 10.1038/s41386-024-01937-9

**Published:** 2024-08-08

**Authors:** Pei X. Luo, Alexandra Serna Godoy, Hannah Cortez Zakharenkov, Nou Vang, Emily C. Wright, Taylor A. Balantac, Sinéad C. Archdeacon, Alexis M. Black, Alyssa A. Lake, Alison V. Ramirez, Lauren E. Lozier, Melvin D. Perez, Irvin Bhangal, Nile M. Desta, Brian C. Trainor

**Affiliations:** 1 Department of Psychology, University of California, Davis, CA USA; 2 Max Planck Florida Institute for Neuroscience, Jupiter, FL USA

**Keywords:** Stress and resilience, Social neuroscience

## Abstract

The hypocretin (Hcrt) system modulates arousal and anxiety-related behaviors and has been considered as a novel treatment target for stress-related affective disorders. We examined the effects of Hcrt acting in the nucleus accumbens shell (NAcSh) and anterodorsal bed nucleus of the stria terminalis (adBNST) on social behavior in male and female California mice (*Peromyscus californicus*). In female but not male California mice, infusion of Hcrt1 into NAcSh decreased social approach. Weak effects of Hcrt1 on social vigilance were observed in both females and males. No behavioral effects of Hcrt1 infused into the adBNST were observed. Analyses of sequencing data from California mice and *Mus musculus* NAc showed that *Hcrtr2* was more abundant than *Hcrtr1*, so we infused the selective Hcrt receptor 2 antagonist into the NAcSh, which increased social approach in females previously exposed to social defeat. A calcium imaging study in the NAcSh of females before and after stress exposure showed that neural activity increased immediately following the expression of social avoidance but not during freezing behavior. This observation is consistent with previous studies that identified populations of neurons in the NAc that drive avoidance. Intriguingly, calcium transients were not affected by stress. These data suggest that hypocretin acting in the NAcSh plays a key role in modulating stress-induced social avoidance.

## Introduction

Social anxiety disorder (SAD) is one of the most prevalent mental disorders across nations [1]. However, existing treatments, such as cognitive behavioral therapy and selective serotonin reuptake inhibitors are only effective in about half of the patients [2]. Social anxiety is more common in women than men, but the mechanisms contributing to sex differences are still under investigation [3, 4]. The hypocretin (Hcrt), or orexin, system has been proposed as a novel treatment target for stress-related mental disorders, including SAD [5] that may also contribute to female vulnerability to stress [6].

One of the key symptoms of SAD is avoidance of social contexts [7]. The Hcrt system is best known for its effects on wakefulness and arousal [8, 9] but preclinical evidence suggests that it also regulates social approach and avoidance [10–13]. For instance, chemogenetic inhibition of Hcrt neurons increased social approach in male rats that were previously exposed to social defeat stress and showed short defeat latency [14]. However, in unstressed male mice, optogenetic inhibition of Hcrt neurons decreased social approach to unfamiliar males [10]. Central manipulations using pharmacological approaches also showed that Hcrt could generate either social approach or avoidance responses [15–18].

One possible explanation for the diverse behavioral outcomes of Hcrt manipulations is that the effects of Hcrt are brain region-specific, similar to serotonin [19] and oxytocin [20] receptors. The neuropeptide Hcrt is present in two isoforms, hypocretin-1 (Hcrt1) and hypocretin-2 (Hcrt2) that are cleaved from the same prepro-hypocretin [21]. There are also two Hcrt G-protein coupled receptors, hypocretin receptor 1 (HcrtR1) and hypocretin receptor 2 (HcrtR2) [22, 23]. HcrtR2 preferentially binds Hcrt2 over Hcrt1 while HcrtR1 binds both peptides with similar affinities [24]. Hcrt neurons are restricted to the lateral, dorsomedial, and perifornical hypothalamus but project widely throughout the brain [25, 26]. The nucleus accumbens (NAc) and bed nucleus of the stria terminalis (BNST) are nuclei where Hcrt could modulate social behavior. Both regions receive direct input from hypothalamic Hcrt neurons [25] and are implicated in regulating social and anxiety-related behaviors. For instance, microinjection of Hcrt1 in the nucleus accumbens shell (NAcSh) of male rats promoted anxiety-like behaviors during open field, light-dark box, and elevated plus maze tests [27]. In another study, intra-BNST infusion of Hcrt1 in male rats reduced social interaction and increased time in the closed arms during elevated plus maze [11].

Overall, prior research on the behavioral effects of Hcrt has been biased towards male rodents [11, 17, 27, 28]. Both human and rodent research revealed sex differences in Hcrt peptide and receptor expression [29–31], suggesting that higher Hcrt activity in females might contribute to the susceptibility to stress exposure [6, 32]. In this current study, we investigated region- and sex-specific effects of Hcrt on behavior in California mice (*Peromyscus californicus*). The California mouse is a monogamous species in which both males and females are aggressive [33, 34]. The high levels of aggression in both sexes have facilitated the study of social defeat in males and females [35, 36]. Social defeat can induce behavioral responses such as social approach, social vigilance, or freezing behavior. First, we investigated the effects of Hcrt1 in the NAcSh and anterodorsal BNST (adBNST) on behavior in a large arena social interaction test with a target mouse confined to a small wire cage. To determine which Hcrt receptors are expressed in these nuclei, we analyzed previously published California mouse bulk sequencing data from the NAc in both sexes [37]. To assess which cell types in the NAc express Hcrt receptors we analyzed single-cell RNA sequencing (scRNA-seq) data of *Mus musculus* NAc [38] and BNST [39]. These data informed our selection of an HcrtR2 antagonist to determine the role of Hcrt in modulating stress-induced avoidance in females. Activation of HcrtR2 enhances neuronal activity within the mesolimbic dopamine system [40, 41], so we used calcium imaging in the NAcSh to track neural activity during behavior in females before and after social defeat exposure. Focal mice were allowed to freely interact with target mice, which allowed for a wider spectrum of behavioral analyses compared to the large arena social interaction test. We correlated calcium transients with avoidance, boxing, and freezing behavior.

## Methods

### Animal

All experiments on California mice were approved by the Institutional Animal Care and Use Committee (IACUC) at the University of California, Davis. Adult (more than 90 days old) male and female California mice from our laboratory colony were co-housed in same-sex groups of 4. Mice were kept on a 16:8 Light:Dark cycle and fed ad libitum (2016 Teklad global 16% protein rodent diets). Sani-chip bedding, cotton nestlets, and Enviro-dri (Newco Distributors) were provided in all cages. Drug infusion and behavioral tests were performed during the dark cycle.

### Cannulation and site-specific injections

Males and females were implanted with 26-gauge bilateral cannula guides aimed at either the NAcSh (A–P: +0.85 mm; M–L: ±1.1 mm; D–V: +5.85 mm) or the adBNST (A–P: +0.0 mm; M–L: ±1.0 mm; D–V: +5.1 mm) and given a 7-day recovery period. During recovery, the animals received subcutaneous injections of carprofen as anti-inflammatory from day 1–3 and were handled daily for 1 min to get habituated to scruffing. During microinjection, 33-gauge internals that projected 1 mm past the guides were used. All animals received 300 nL bilateral infusions lasting 2 min and the internals were left in for an additional 30 s. Stress-naïve mice were randomly assigned to receive either saline (vehicle) or Hcrt1 (300 ng or 30 ng in the NacSh; 300 ng in the adBNST; OrexinA, Tocris #1455). The dosage selection was based on previous rodent studies [11, 27].

A separate cohort of female mice were exposed to social defeat stress 1 week prior to surgery. Mice were implanted with guides aimed at the NAcSh and one week later received either saline (vehicle) or 13 µg HcrtR2 antagonist (TCS OX2 29, Tocris #3371) before behavior testing. Mice with guides aimed at the adBNST received either 20% DMSO (vehicle) or 0.3 µg HcrtR1 antagonist (SB 334867, Tocris #1960). The dosage selection was based on previous rodent studies [27, 42, 43]. Injection volumes were 300 nL. A social interaction test was performed 20 min following the infusion. Brains were collected for histology to confirm successful cannula placement.

### Social interaction test

The social interaction test consisted of 3 phases, each lasting 3 min [44]. Mice were introduced into an empty arena (89 × 63 x 60 cm) and allowed to freely explore during the open field phase. During the acclimation phase, an empty wire cage was placed against one side of the arena for habituation. For the social interaction phase, a same-sex unfamiliar target mouse was placed into the wire cage. Distance traveled, time in the center zone (located 14 cm from the sides), and time that the focal mouse spent within the interaction zone (within 8 cm of the wire cage) were recorded and analyzed using AnyMaze. We operationally define the time spent in the interaction zone (with the target mouse present) as social approach, with the understanding that time spent in the zone could be modulated by neural circuits promoting approach or avoidance. The time that the focal mouse spent outside of the interaction zone with its head oriented towards either an empty cage or target mouse was defined as vigilance and scored manually.

### Social defeat stress

Mice assigned to social defeat were placed in the homecage of an aggressive same-sex mouse [36]. Each defeat episode lasted 7 min or until the resident attacked the focal mouse 7 times, whichever occurred first. The intruder mice were immediately returned to their homecage following the defeat. This procedure was performed on three consecutive days.

### Calcium imaging of the NAc

Adult females received an injection of AAV9.Syn.GCaMP6f (Addgene 100843-AAV9) at a rate of 100 nL/min for a total volume of 300 nL in the NAc shell (A–P: +0.85 mm; M–L: ±1.1 mm; D–V: −5.85 mm) and the needle was left in place for 10 min before it was withdrawn. Each mouse was implanted with a single optical fiber (Doric) with 2.5 mm core and 0.66 NA threaded through a ceramic ferrule was implanted at the injection site and the ferrule was secured to the skull with C&B Metabond (Parkell) and dental cement (Fig. S1). Mice were housed two per cage with a clear, perforated acrylic divider. Dividers prevented cagemates from damaging one another’s implant while still allowing for auditory, tactile, and olfactory contact.

Mice recovered for 3 weeks before undergoing three consecutive days of patch cord habituation in which the patch cord was gently coupled to their optical fiber implant. The mouse then explored a novel cage for 10 min. One day following the last habituation, each mouse was tested in a small arena social interaction test [45]. We used a within-subjects design (before and after social defeat) based on previous data showing that this approach can have greater statistical power than a between-subjects design [46]. The focal mouse was placed into an empty area (51 × 25.4 × 76 cm) attached to a small box (13 x 10 x 18 cm) with a sliding door for 6 min. Photometry recording was performed with an isosbestic channel of 405 nm and an excitatory channel of 470 nm, both set to 50 μW output. A 10 min acclimation period to the novel arena was used to perform initial bleaching of GCaMP. Next, an unfamiliar, non-aggressive adult female target mouse was introduced into the arena through the sliding door. Mice were allowed to interact for 3 min. The target mouse was removed and then a sexually experienced, female target mouse with previous experience winning aggressive interactions was introduced into the arena for 3 min. Two days later mice were exposed to three episodes of social defeat and then tested again with new target mice one week after the last episode of defeat. After testing, brains were collected to confirm viral expression and placement of the fiber.

A total of seven female California mice had fibers correctly placed in the NAcSh with robust GCaMP expression. Behavior was scored by a trained observer using BORIS. Using BORIS, we slowed videos down to identify the exact frame in which behaviors began, which allowed us to precisely identify behaviors such as approach or avoidance. Avoidance was defined as the 2 s following the moment the focal mouse turned away from the target mouse to move away. We observed only a few instances in which target mice approached focal mice and we did not have a large enough sample for analysis. This result was surprising because we observed social approach behavior in a similar experiment of male California mice [45]. We hypothesize that even though we performed habituation to patch cord attachment, the handling involved with this procedure may have impacted female behavior. Future studies will evaluate whether delaying behavioral testing (20 min instead of 10 min) after patch cord attachment results in more instances of social approach in female California mice. Boxing was defined as when the focal mouse extended its forearms towards the target mouse, which occurred when the target mouse approached the focal mouse. Freezing was defined as the focal mouse remaining immobile for at least 1 s. We performed repeated measures ANOVA to examine for effects of stress and target mouse status on these behaviors.

### Statistical analyses

Behavioral data analyses were performed in RStudio. The Shapiro-Wilk’s test was used to test for data normality and the Fligner-Killeen test was used to assess homogeneity of variance. For Hcrt1 infusions into the NAcSh 1, two-way ANOVA (sex*treatment) followed by planned comparisons were used to analyze normally distributed behavioral data. For Hcrt antagonists and experiments in the BNST, *t*-tests were to compare drug treatments with controls. Vigilance data were log-transformed to correct for heterogenous variance. Cohen’s d was calculated to reflect effect sizes of the significant differences. Animals with misplaced cannula guides were included in the analysis as anatomical controls when the sample size was >6. Estrous cycle was determined via vaginal lavage and no main effects or interaction effects (estrous*treatment) of estrous stage were detected, consistent with prior studies in California mice using the social interaction test [36, 47].

We used previously published California mouse bulk sequencing data (Williams et al. 2022, Bioproject: PRJNA700778) to analyze the abundance of *Hcrtr1* and *Hcrtr2* in the NAc (male and female), medial prefrontal cortex (female), and ventral tegmental area (female). Males and females were randomly assigned to defeat stress and mice were tested in a social interaction test 2 weeks later. The next day mice were euthanized and punch samples of the NAc, ventral tegmental area (VTA), and medial prefrontal cortex (mPFC) were collected. Within each brain area, we used repeated measures (transcript type) ANOVA to analyze reads per kilobase per million mapped reads (RPKM) and test for sex and stress effects. We used Spearman rank correlations to test if expression of each transcript was correlated with social approach, approach to an empty cage, or behavior in the open field test.

Single-cell sequencing data were analyzed in RStudio using Seurat v4.0.4 [48]. We accessed published data [38] from 11 adult male *Mus musculus* NAc single-cell RNA-seq data containing 47,576 total cells, organized into 21,842 neuronal and 25,734 non-neuronal cells from GEO: GSE118020 along with cluster identities. A total of 57 *Hcrtr1+* and 464 *Hcrtr2+* cells were subsetted, analyzed for cell type composition, and visualized using the DotPlot function. The same approach was applied to the *Mus musculus* BNST snRNA-seq data containing 76,693 neurons across seven adult female and eight adult male biological replicates ([39] from GEO:GSE126836).

Photometry data was analyzed using a custom Python script [45], down sampled to 30 samples per second to match the 30 frames per second video framerate. 405 nm was used as the isosbestic wavelength and 470 nm as the excitatory wavelength. To correct for motion artifact and bleaching of the fluorophore, the 405 nm signal was fit to a biexponential model and subtracted from the excitatory output such that ΔF/F = [100*((470 nm signal—fitted signal)/fitted signal)]. *Z*-scores were calculated using the formula: z-score = ((ΔF/F − mean ΔF/F of baseline period)/standard deviation of ΔF/F of baseline period). The baseline period was −8 to −6 s before the behavior onset. Area under the curve (AUC) was calculated using the trapezoidal method for three different time periods: baseline (as defined in relation to the *z*-score calculation), before (the 2 s preceding the behavioral event onset), and after (the 2 s following the behavioral event onset). Linear mixed-effects models were used to determine the effects of stress condition (pre-stress vs post-stress), social condition (non-aggressive target vs aggressive target), and time point (baseline vs before vs after) using the statsmodels package [49] in Python using the formula:

$${{{{\rm{AUC}}}}}= \, 	 {{{\rm{stress}}}}\, {{{\rm{condition}}}}+{{{\rm{social}}}}\,{{{\rm{condition}}}}+{{{\rm{time}}}}\,{{{\rm{point}}}}+{{{\rm{stress}}}}\,{{{\rm{condition}}}}^{\ast }{{{{\rm{social}}}}\,{{{\rm{condition}}}}}\\ 	 +{{{\rm{stress}}}}\,{{{\rm{condition}}}}^{\ast }{{{\rm{time}}}}\,{{{\rm{point}}}}+{{{\rm{social}}}}\,{{{\rm{condition}}}}^{\ast }{{{\rm{time}}}}\,{{{\rm{point}}}}\\ 	 +{{{\rm{stress}}}}\,{{{\rm{condition}}}}^{\ast }{{{\rm{social}}}}\,{{{\rm{condition}}}}^{\ast }{{{\rm{time}}}}\,{{{\rm{point}}}}+(1|{{{\rm{mouse}}}})$$

## Results

### Hcrt1 infusion in the NAcSh reduces social approach in females

**Fig. 1:**
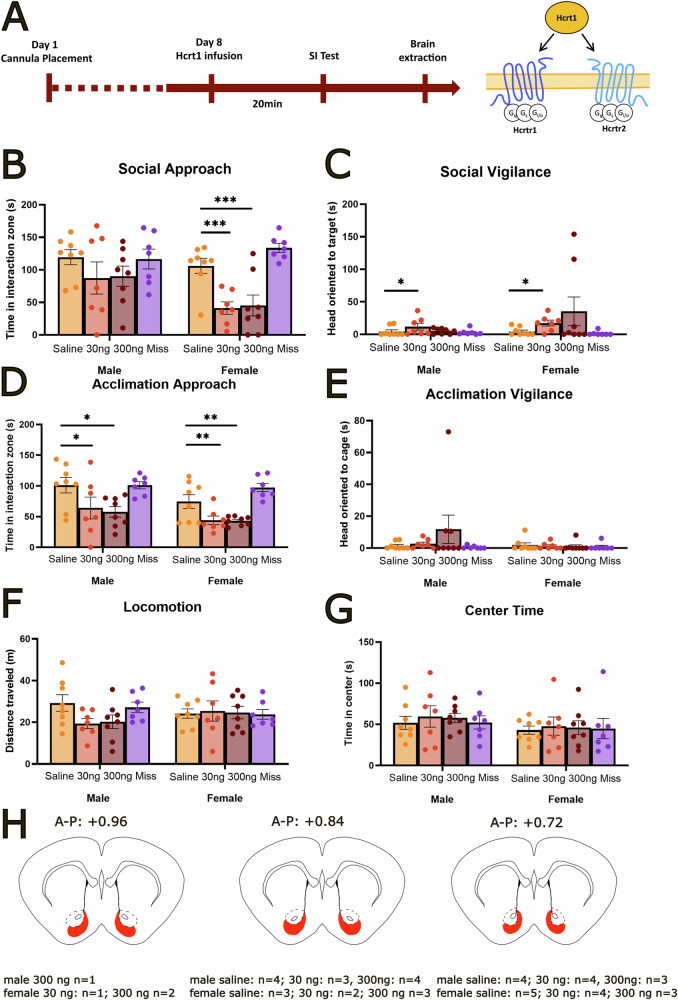
Hcrt1 infusion in the NAcSh reduced social approach and vigilance in stress-naïve female California mice. Timeline of experiment and mechanism of action for Hcrt1 (**A**). Infusion of 30 ng or 300 ng Hcrt1 in the NAcSh reduced social approach in females, but not males (**B**). Infusion of 30 ng but not 300 ng Hcrt1 increased social vigilance in females and males (**C**). Intra-NAcSh infusion of 30 ng and 300 ng Hcrt1 decreased approach of a novel empty cage during the acclimation phase in both sexes (**D**) but had no effects on vigilance (**E**). Infusion of Hcrt1 in the NAcSh had no effect on distance traveled (**F**) or time spent in the center (**G**) during the open field phase. Schematics representing injection sites (red shading) of successful cannula placement in the NAcSh (**H**). *p < 0.05, **p < 0.01, ***p < 0.001 vs saline. Group n’s Male/saline *n* = 8, male/Hcrt1 30 ng *n* = 7, male/Hcrt1 300 ng *n* = 8, male/miss *n* = 7, female/saline *n* = 8, female/Hcrt1 30 ng *n* = 7, female/Hcrt1 300 ng *n* = 8, female/miss, *n* = 7.

To determine if Hcrt receptors modulated social approach (Fig. 1A), we infused Hcrt1 into the NAcSh of unstressed male and female mice. We observed that Hcrt1 had stronger effects on social approach in females than males (Fig. 1B, sex*treatment *F*_3,52_ = 8.78, *p* < 0.001). In females, microinjection of 30 ng (planned comparison, *p* < 0.001, *d* = 2.18) or 300 ng (planned comparison, *p* < 0.001, *d* = 1.54 for 300 ng) of Hcrt1 in the NAcSh decreased social approach to a novel same-sex target mouse compared to saline (Fig. 1B). There were no effects of Hcrt1 on social approach in males. There was a main effect for Hcrt1 infusion to increase social vigilance (Fig. 1C, *F*_3,52_ = 3.55, *p* < 0.05). Although the sex*treatment term was not significant, there was a larger effect size of the 30 ng dose versus saline in females (*d* = 1.2) than in males (*d* = 0.8). The 300 ng was not different from saline in either sex.

Infusion of Hcrt1 also reduced approach to a novel empty cage during the acclimation phase (Fig. 1D, main effect of Hcrt, *F*_1,52_ = 5.53, *p* < 0.05). In both males (planned comparison, *p* < 0.05, *d* = 0.90 for 30 ng; *p* < 0.05, *d* = 1.43 for 300 ng) and females (planned comparison, *p* < 0.01, *d* = 1.15 for 30 ng; *p* < 0.01, *d* = 1.36 for 300 ng) Hcrt1 infusions reduced approach to the empty cage compared to saline. No differences were observed in vigilance behavior during the acclimation phase for males or females (Fig. 1E). No differences were observed in total distance traveled (Fig. 1F) and time spent in the center of the open field phase (Fig. 1G). Animals with misplaced cannula guides (Fig. 1H) were not different from controls.

### Inhibition of HcrtR2 in the NAcSh decreases social avoidance in stressed females

**Fig. 2:**
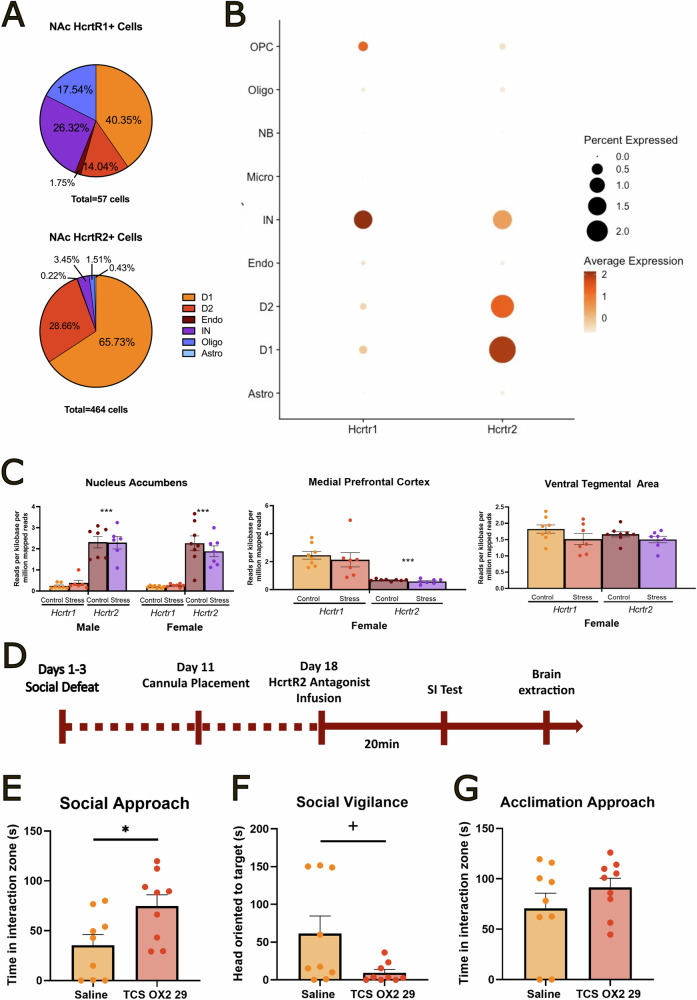
Selective HcrtR2 antagonist infusion in the NAcSh increased social approach but not social vigilance behaviors in stressed female California mice. In male *Mus*, NAc cells were organized into 8 cell types: astrocytes (Astro), oligodendrocytes (Oligo), endothelial cells (Endo), interneurons (IN), dopamine receptor 1-expressing cells (D1), and dopamine receptor 2-expressing cells (D2). *Hcrtr2* is primarily expressed in the dopamine D1 receptor medium spiny neurons and interneurons, whereas *Hcrtr1* is primarily expressed in the D1 and D2 medium spiny neurons (**A**). A Dotplot shows that *Hcrtr1* and *Hcrtr2* have distinct expression patterns across all cell types in male *Mus*. *Hcrtr1* has the highest average and percent expression in the interneurons, whereas *Hcrtr2* has the highest average and percent expression in D1 neurons (**B**). In the nucleus accumbens but not medial prefrontal cortex or ventral tegmental area *Hcrtr2* was more abundant than *Hcrtr1* (**C**). Timeline of experiment in stressed female California mice and schematic of the mechanism of action for selective HcrtR2 antagonist TSC OX2 29 (**D**). Infusion of TSC OX2 29 in the NAcSh of female California mice previously exposed to social defeat stress increased social approach (**E**) but had no effects on social vigilance behavior (**F**), behaviors during the acclimation phase (**G**). *p < 0.05, +*p* = 0.06 vs. saline. ***p < 0.001 vs. *Hcrtr1*. Group n’s: gene expression; male/control *n* = 7, male/stress *n* = 6, female/control *n* = 8, female/stress *n* = 7, pharmacology; female/saline *n* = 9, female/OX2 29 *n* = 9.

Social defeat reduces social approach in female California mice [36, 44], so we sought to inhibit Hcrt receptors to determine if this system mediates effects of stress on behavior. To determine which receptors to target, we analyzed published RNA sequencing data [38] from the NAc to determine the relative abundance of *Hcrtr1* and *Hcrtr2* cells. Adult male *Mus musculus* NAc cells were grouped into eight different clusters: astrocytes, oligodendrocytes, endothelial cells, interneurons, dopamine receptor 1-expressing cells, dopamine receptor 2-expressing cells, and oligodendrocyte progenitor cells. Out of 47,576 NAc cells, 57 expressed *Hcrtr1*, and 464 expressed *Hcrtr2*. The majority of *Hcrtr1+* neurons were D1 medium spiny neurons and interneurons, while the majority of *Hcrtr2+* neurons were D1 medium spiny neurons (Fig. 2A). *Hcrtr1+* and *Hcrtr2+* also had distinct expression patterns across cell types. *Hcrtr1* had the highest percent expression (1.5%) and average expression (2.1 standard deviation above mean expression) within interneurons, whereas *Hcrtr2* had the highest percent (2.1%) and average expression (2.0 standard deviation above mean expression) within D1 medium spiny neurons (Fig. 2B). Since activation of oxytocin receptors in the NAc drive social approach behaviors (Williams et al. 2020) we examined whether *Hcrtr2* was expressed in the same cells as *Oxtr* (Fig. S2). In both D1 neurons (Fig. S2A) and interneurons (Fig. S2B) *Hcrtr2* and *Oxtr* were expressed in different cells.

Consistent with the *Mus* data, bulk RNAseq data from California mice showed that *Hcrtr2* was almost 10-fold more abundant than *Hcrtr1* in the NAc (Fig. 2C, transcript F_1,13_ = 140.48, *p* < 0.001). The preferential expression of *Hcrtr2* over *Hcrtr1* was limited to the NAc and not present in the VTA or mPFC. There were no sex differences and no effect of stress on either transcript. In female mPFC *Hcrtr1* was more abundant than *Hcrtr2* (Fig. 2C, transcript F_1,13_ = 34.25, *p* < 0.001) and there was no effect of defeat stress. There were no differences in transcript abundance or effects of stress in female VTA samples (Fig. 2C). Importantly, there were no differences in total reads per sample across comparison groups within the NAc, mPFC, or VTA. There were no significant correlations between *Hcrtr1* or *Hcrtr2* with any behaviors measured in the social interaction test. Since we had strong evidence that *Hcrtr2* was more abundant than *Hcrtr1* in the NAc, we chose to inhibit HcrtR2 in females exposed to social defeat (Fig. 2D).

Stressed female California mice treated with a selective HcrtR2 antagonist infusion in the NAcSh showed increased social approach (Fig. 2E, *t*_16_ = 2.49, *p* < 0.05, *d* = 1.18) and a non-significant trend for decreased vigilance (Fig. 2F, *t*_16_ = 2.26, *p* = 0.06, *d* = 1.12) There were no differences between groups during the open field and acclimation phases (all *p*’s > 0.05, Figs. 2G, S3A–C).

### Hypocretin ligands in the adBNST do not affect social approach or vigilance

**Fig. 3:**
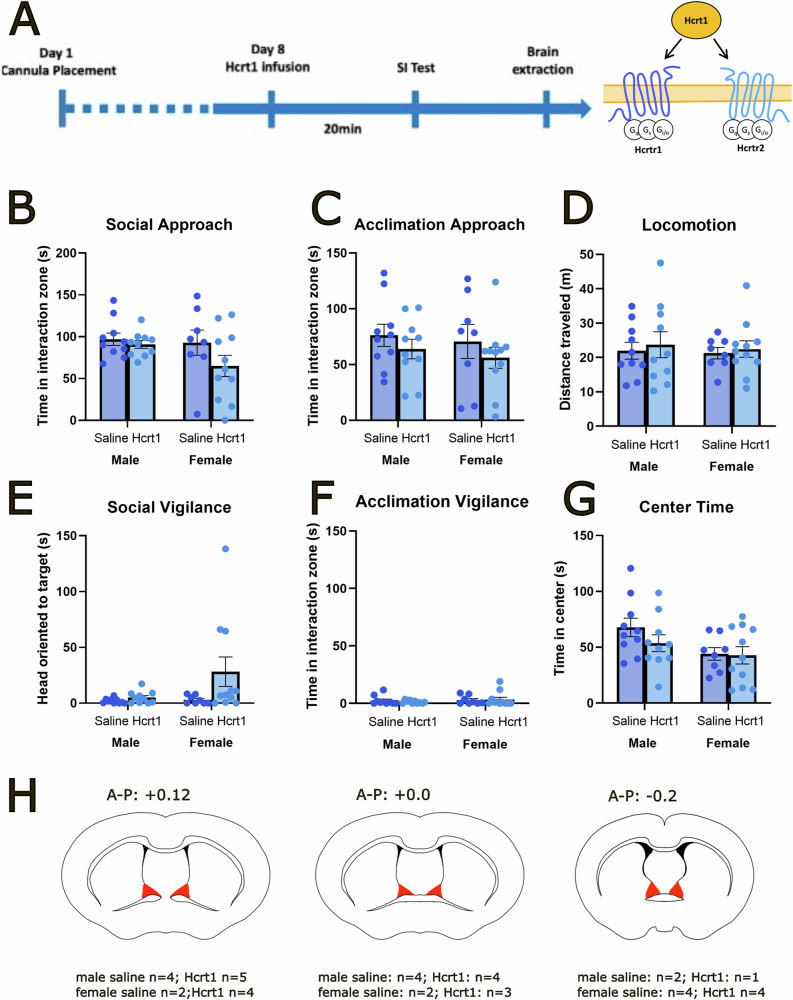
Hcrt1 infusion in the adBNST had no behavioral effects during the social interaction test in male or female California mice. Timeline of experiment and schematic of mechanism of action for Hcrt1 (**A**). Infusion of 300 ng Hcrt1 in the adBNST in male and female California mice had no effects on social approach (**B**), acclimation approach (**C**), locomotion (**D**), social vigilance (**E**), acclimation vigilance (**F**) or center time during the open field (**G**). Schematic representing injection sites (red shading) of successful cannula placement in the NAcSh (**H**). Group *n*’s Male/saline *n* = 10, male/Hcrt1 *n* = 10, female/saline *n* = 8, female Hcrt1 *n* = 11.

To determine if effects of Hcrt1 on behavior were site-specific, we infused Hcrt1 into the adBNST. Intra-BNST infusion of 300 ng Hcrt1 did not affect behaviors during social interaction, acclimation, or the open field phase in male or female California mice (Fig. 3B–G). Infusion of selective HcrtR1 antagonist also had no effects in stressed females during the 3-phase social interaction test (Fig. S4D–I). We also analyzed previously published RNA sequencing data from the BNST [39] to determine which cell types expressed Hcrt receptors. In adult *Mus* BNST, *Hcrtr2* was more abundant than *Hcrtr1* (Fig. S4A). In male *Mus*, 14.09% of BNST cells express *Hcrtr2*, 2.94% express *Hcrtr1* and only 0.49% express both receptors. In female *Mus*, 13.35% BNST cells express *Hcrtr2*, 1.49% express *Hcrtr2*, and only 0.28% express both receptors. Adult *Mus* neurons were grouped into 41 clusters based on Welch et al., 2019. Both receptor types were expressed across multiple neuron types and had similar expression patterns between males and females (Fig. S4B). In the anterior BNST three cell types (*Cyp26b1*, *Sin*, and *Col5a3*) had the highest levels of *Hcrtr2* expression in both males and females. In contrast, *Hcrtr1* had the highest average expression (2.5 standard deviations above mean expression) the highest percent expression (16.73% of *Esr2* cells for males and 7.91% for females) in the *Esr2* cluster in the principal subdivision of BNST.

### Calcium imaging in the Nucleus accumbens shell

**Fig. 4:**
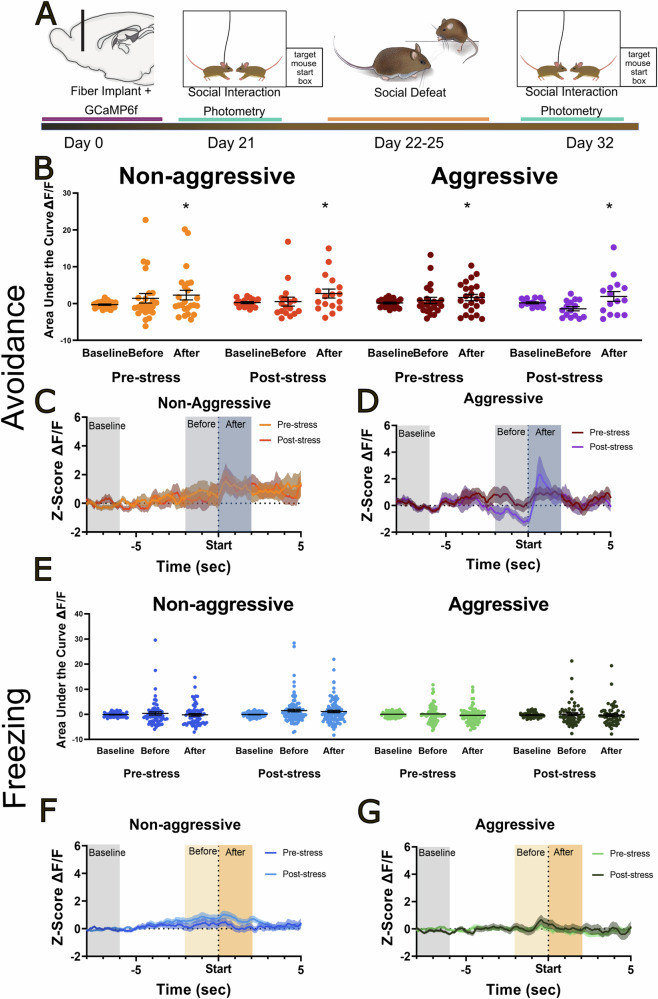
Increased nucleus accumbens shell activity coincides with behavioral avoidance. Experimental timeline for fiber photometry observations of GCaMP6 in the NAcSh of female California mice (**A**). GCaMP6f signals were significantly stronger in the 2 s period following the initiation of avoidance of a target mouse (**B**–**D**). No changes in GCaMP6f signals were observed following bouts of freezing (**E**–**G**) in the presence of non-aggressive or aggressive target mice. *p < 0.05 versus baseline. Seven females were used for these analyses.

Finally, we used calcium imaging to assess changes in neural activity in the NAc shell during social interactions (Fig. 4A). In a previous photometry study conducted on male California mice [45], males engaged with target mice with aggression and bouts of freezing. In the present study on female California mice, we observed bouts of boxing and avoidance. Neural activity in the NAc increased compared to baseline immediately following the initiation avoidance (Fig. 4B–D, *β* = 2.58, *z* = 2.17, *p* < 0.05) or boxing (Fig. S5B–D, *β* = 2.80, *z* = 2.39, *p* < 0.05). There were no significant effects of target mouse status (non-aggressive or aggressive) or stress and there were no significant interaction terms. There was no significant effect of stress or target mouse on the frequency of avoidance (Fig. S6A). For boxing there was a non-significant interaction between stress and target mouse, with boxing (Fig. S6B stress*target *F*_1,6_ = 5.04, *p* = 0.06) but paired t-tests were not significant. Stress increased freezing behavior (Fig. S6C, stress *F*_1,6_ = 9.34, *p* = 0.02). While the target*stress comparison was not significant, there was a larger effect size of stress in the presence of non-aggressive target mice (*t*_6_ = 3.69, *p* = 0.01, *d* = 2.3) compared to aggressive target mice (*t*_6_ = 1.86, *p* = 0.11, *d* = 0.6). No changes in neural activity were observed during the expression of freezing behavior (Fig. 4E–G).

## Discussion

The effects of Hcrt1 on social approach in the NAc were limited to female but not male California mice. The same doses of Hcrt1 that reduced social approach in females also reduced approach to a novel empty cage in both sexes. Inhibition of HcrtR2 in stressed females increased social approach, suggesting that stress may increase Hcrt release in the NAcSh. Effects of Hcrt receptors were site-specific as Hcrt receptor ligands had no behavioral effects in the adBNST. In the central nervous system, HcrtR2 drives excitatory Gq signaling [50] and enhances neuronal activity across many brain regions [40, 41]. When we used calcium imaging to track neural activity in the NAcSh, we observed increased activity when focal mice engaged in avoidance or boxing. No changes in calcium signals were observed at the initiation of freezing behavior. Although we did not measure Hcrt release directly, we speculate that these results are consistent with the hypothesis that activation of HcrtR2 in the NAcSh may facilitate increased neural activity during the expression of social avoidance.

We observed that *Hcrtr2* was significantly more abundant than *Hcrtr1* in the NAc in California mice and that activation of HcrtR2 in the NAc shell was necessary for stress-induced decreases in social approach. This result is consistent with previous work showing that inhibition of HcrtR2 in the NAc shell of male rats blocked stress-induced changes in behavior in the elevated plus maze and open field test [27]. We observed no effects of defeat stress on *Hcrtr2* or *Hcrtr1* expression in either male or female California mice, which suggests that defeat stress does not alter the abundance of receptors in the NAc. Thus HcrtR2-dependent social avoidance in stressed females is likely driven by increased Hcrt release. Optogenetic stimulation of Hcrt neurons in male mice drove increased corticosterone secretion and anxiogenic responses in an open field test [51] while Hcrt knockdown blocked behavioral responses in a rat panic model [17]. While we found that Hcrt ligands had no impact on locomotor activity in the open field phase of the test, Hcrt1 infusions in the NAcSh reduced approach to a novel empty cage in male and female California mice, suggesting that Hcrt can have anxiogenic effects across a variety of contexts.

An intriguing question is why Hcrt1 reduced approach to an empty cage whereas previous California mouse studies reported that defeat stress does no affect approach to an empty cage [37, 52]. One possible explanation for the difference between defeat stress and Hcrt1 infusion on approach to an empty cage is that defeat may only facilitate Hcrt release in social contexts. Calcium imaging is an ideal approach to assess context-dependent activity of Hcrt neurons because of its fine temporal resolution. In male mice, Hcrt neurons showed the strongest increases in activity following exposure to predator odors [53], although female odors also generated significant increases in activity. Similarly, restraint stress robustly activated hypothalamic Hcrt neurons in male mice [54]. While Hcrt release is thought to be activity-dependent, these studies did not assess Hcrt release directly. A human Hcrt receptor was genetically modified to create a Hcrt sensor that produced a fluorescent signal in the presence of high concentrations of Hcrt [55]. This sensor could detect Hcrt release in the insular cortex during tail suspension in mice, an aversive experience. Future studies could use these tools to assess the extent to which Hcrt neurons are activated in social and non-social contexts.

While we did not measure Hcrt release directly, we used GCaMP imaging in the NAcSh to evaluate neural activity during social interactions. Calcium transients measured by fiber photometry indicated that neural activity in the NAcSh tracked the expression of avoidance regardless of stress exposure or the status of the target mouse. Previous work suggests that distinct populations of neurons within the NAcSh drive avoidance and approach. Optogenetic stimulation of ventral NAcSh (which was the target for our pharmacology and fiber photometry studies) drives aversion in a real-time place preference assay in male C57Bl6/J mice [56]. In contrast, activation of dynorphin neurons in the dorsal NAcSh drove place preference. In previous work, we observed that oxytocin receptor in the NAc core promotes social approach in males and females [57]. We observed that *Hcrtr2* and *Oxtr* were expressed in different neurons, suggesting that there are distinct cell populations in the NAc that can drive avoidance or approach. Our finding that GCaMP transients increased as mice engaged in avoidance is consistent with previous findings that social defeat increases indirect markers of neural activity in the NAcSh of female California mice exhibiting social avoidance [36]. Infusion of D1 agonist into the ventral NAcSh reduced social approach in unstressed female but not male California mice [58], which mimicked our effects of Hcrt1 infusions in males and females. Together these observations suggest that D1/Hcrt interactions could be important for driving social avoidance. This may be an important factor in why Hcrt1 infusion had sex-specific effects in the absence of sex differences in receptor expression. Dopamine D1 receptor neurons are often treated as a unitary cell type, however, findings from male C57Bl6/J mice suggest that there are important subpopulations of D1 neurons with different functions.

Male C57Bl6/J mice that exhibited social avoidance after exposure to defeat stress had decreased excitatory input onto NAc D1 neurons and increased excitatory input into D2 neurons [59]. When D1 neurons were stimulated in stressed mice using optogenetics, social approach increased, in apparent opposition to our previous findings in California mice. One possible explanation for these outcomes comes from recent data demonstrating heterogeneity within NAc D1 cell types. An activity-dependent tagging study in male C57Bl6/J mice found a distinct subpopulation of D1 medium spiny neurons that was activated by aversive contexts [60]. This subpopulation of D1 neurons expressed *Tac1* and *Calb1*, and when these cells were activated by excitatory optogenetic stimulation they drove conditioned place aversion. An important gap in the field is understanding how NAc cell types modulate approach and avoidance behaviors in females, as the majority of work in the field has focused on male rodents.

In contrast to our experiments in the NAcSh, the same doses of Hcrt1 had no behavioral effects when infused in the anterodorsal BNST. A previous study identified anxiogenic effects of HcrtR1 in the BNST using a male rat model of panic [17]. However, these behavioral effects were dependent on chronic inhibition of GABA synthesis to yield vulnerability to panic-like attacks, and the HcrtR1 antagonist was injected in the posterior region of BNST (whereas we targeted anterior BNST). A different study in male rats reported that 100 ng of hypocretin-A (similar to our 30 ng dose) infused into the BNST reduced time spent in the open arms of an elevated plus maze and social approach [11]. An important difference between the rat social interaction test and our studies is that the rat test allowed for free social interaction whereas our pharmacology analyses were performed with target mice confined to a small wire cage. Each approach has advantages and disadvantages. Confining a target mouse to a cage allows for the quantification of social approach and vigilance in a standardized approach. In contrast, allowing a focal animal to interact with a target mouse increases variability (as the behavior of the target mouse cannot be controlled) but allows for the quantification of a wider array of behaviors. In addition, Lungwitz et al. [11] targeted both anterodorsal and anteroventral BNST, whereas we limited our injection site to only anterodorsal BNST. The BNST has many subregions with different subtypes [61]. Activation of different subregions within the BNST could produce divergent behavioral effects [60]. Further study should take anatomical differences into account and determine if the behavioral effects of Hcrt receptors in the BNST are context-dependent or dependent on Hcrtr2.

We identified important sex differences in how Hcrt acting in the NAcSh modulates social avoidance and vigilance. Sex differences in the Hcrt system have been reported in both the human and rodent literature. In women but not men diagnosed with depression, Hcrt1 immunoreactivity was increased in the hypothalamus versus healthy controls [31]. In female rats, restraint stress increased hypothalamic Hcrt expression and increased Hcrt/c-fos colocalizations compared to males [32]. HcrtR1 expression in the hypothalamus [62] and HcrtR2 expression in the paraventricular nucleus [30] were higher in female rats than males. Based on these findings it has been hypothesized that increased Hcrt receptor activation contributes to increased female stress responses [6]. One possible mechanism for these differences is via gonadal hormone regulation, as in humans the promoter region of the Hcrt1 gene contains estrogen response elements that facilitate transcription in the presence of estradiol [63]. Our results reinforce the notion that Hcrt exerts sex-specific effects on behavior and that future studies should take a circuit-specific approach towards studying Hcrt regulation of behavior.

## Supplementary information

Supplementary Figures

## Data Availability

All scripts for fiber photometry analyses are deposited at github.com/bctrainorlab/Wrightetal_2023. Data from figures are available upon request.
