# Supplementary material for: Hypocretin in the nucleus accumbens shell modulates social approach in female but not male California mice
Source: Neuropsychopharmacology. 2024 Aug 8;49(13):2000–10. doi: 10.1038/s41386-024-01937-9 (PMC11480414; doi:10.1038/s41386-024-01937-9)
Supplement: Supplementary file 1 — Supplementary Figures [file 41386_2024_1937_MOESM1_ESM.docx]

Supplementary Figures


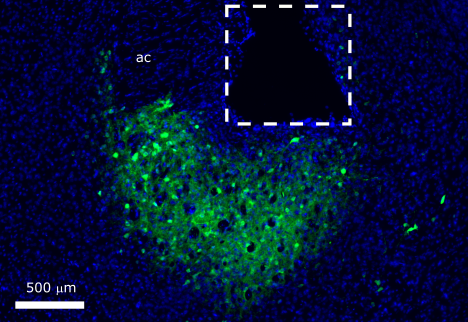


Supplementary Fig. 1: Histology of fiber placement in the nucleus accumbens. Green cells represent GCaMP6 positive cells. Magenta box represents fiber placement. Ac: anterior commissure.


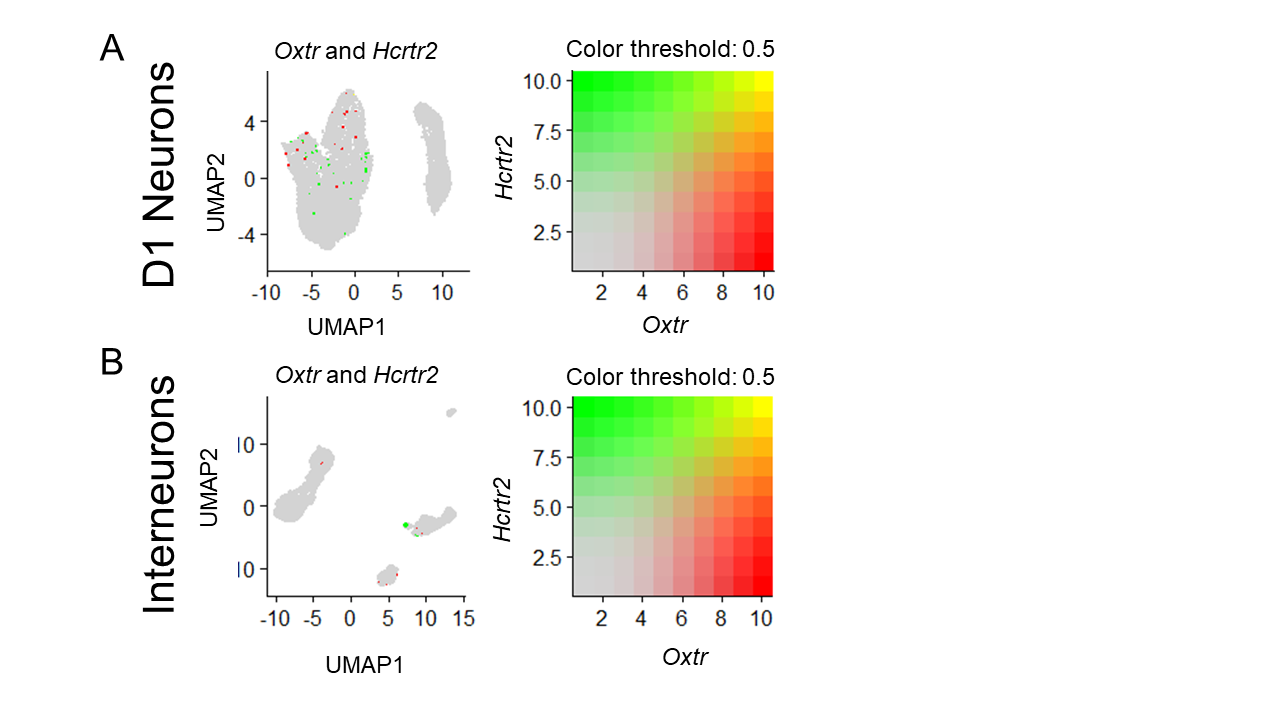


Supplementary Figure 2: Expression of Oxtr and Hcrtr2 in D1 neurons (A) and interneurons (B). Uniform manifold approximation and projection (UMAP) shows the distributions of cells for each cell type. The color of each dot represents the relative expression of *Oxtr* (red) and *Hcrtr2* (green). A yellow dot would represent a cell expressing both *Oxtr* and *Hcrtr2*. In both D1 neurons and interneurons, *Oxtr* and *Hcrtr2* are largely expressed in different cells.


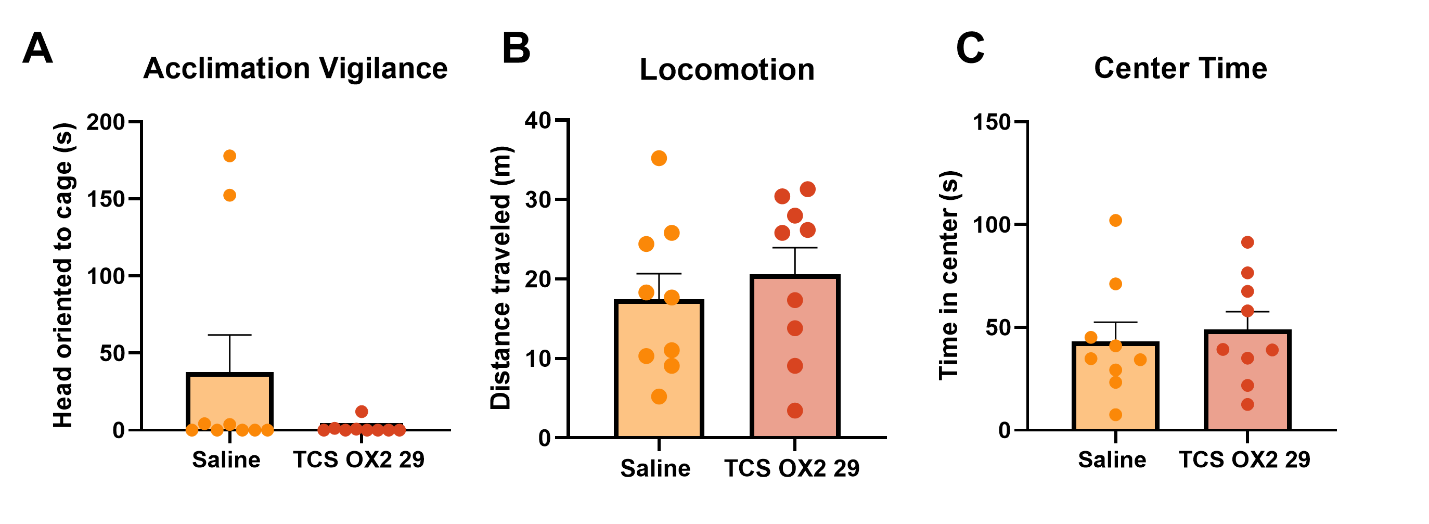


Supplementary Figure 3: There were no effects of hypocretin antagonist (TCS OX2 29) on vigilance during acclimation (A), locomotion during the open field stage (B) or time spent in the center during the open field (C).

*
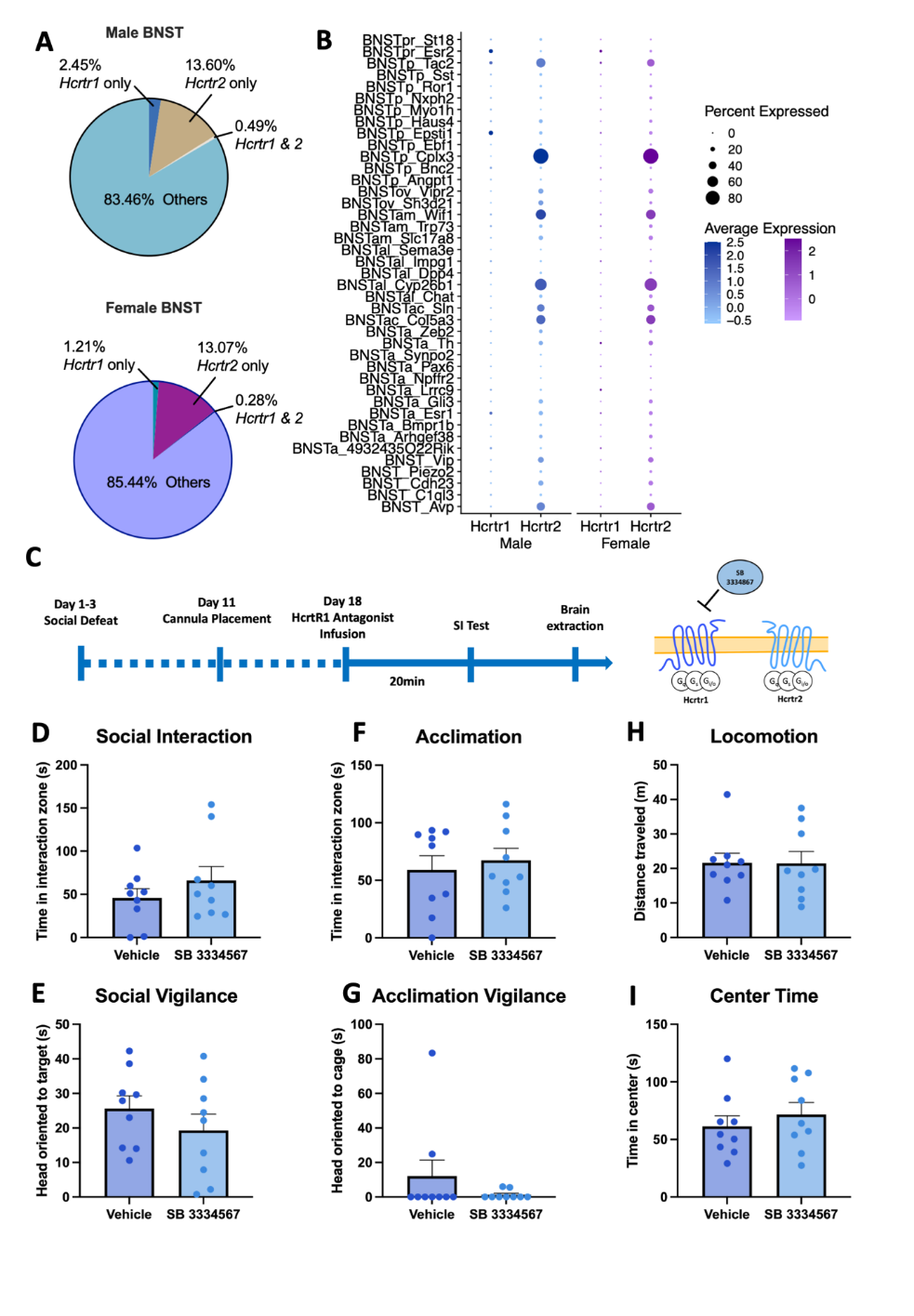
*

Supplementary Figure 4. Selective HcrtR1 antagonist infusion in the adBNST did not affect social interaction behaviors in stressed female California mice. In both male and female *Mus musculus* BNST, *Hcrtr2* is more abundant than *Hcrtr1* and only a small percentage of the cells express both receptor types (A). Both receptor types were expressed across multiple neuron types and had similar expression patterns between males and females (B). Timeline of experiment in stressed female California mice and schematic of mechanism of action for selective HcrtR1 antagonist SB 3334867 (C). Infusion of SB 3334867 in the adBNST of females California mice previously exposed to social defeat stress had no effects on behaviors during the 3-phase social interaction test (D-I).

*
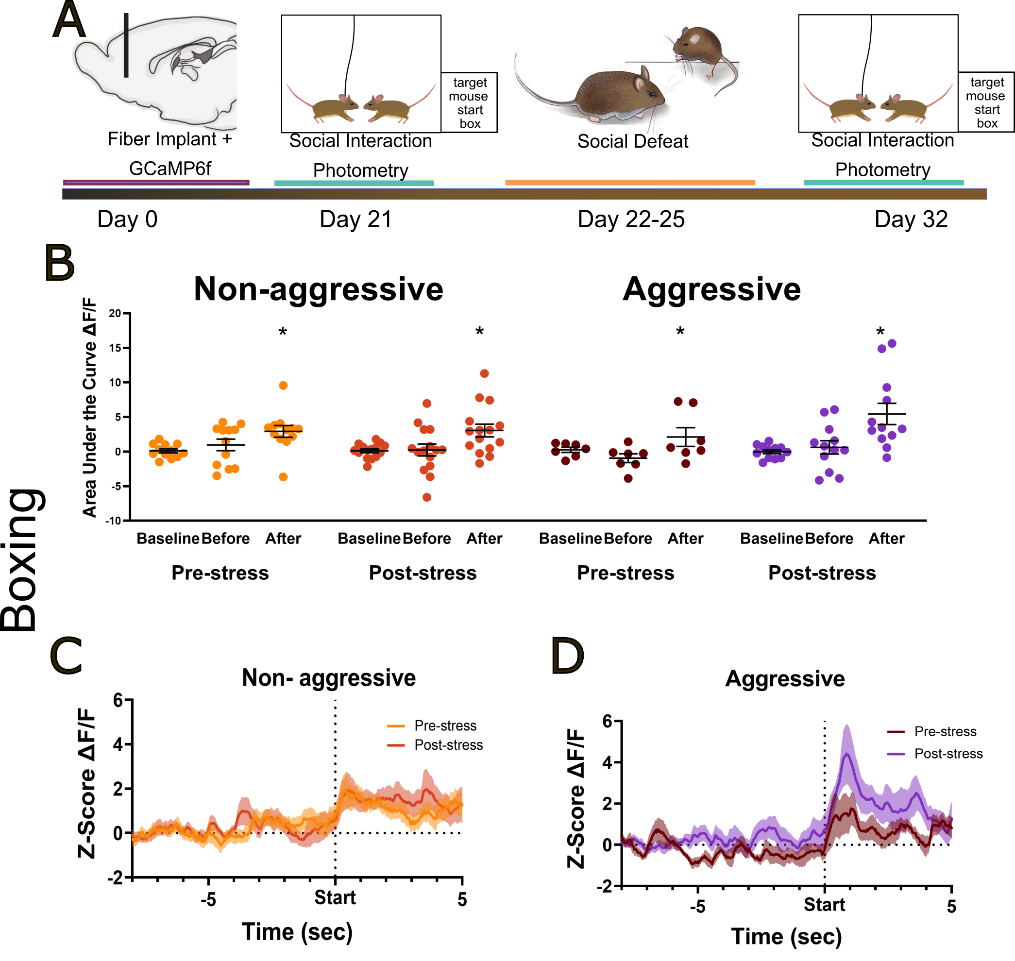
*

Supplementary Figure 5: Behavioral analyses from GCaMP imaging in the nucleus accumbens. Experimental timeline for fiber photometry observations of GCaMP6 in the NAcSh of female California mice (A). GCaMP6f signals were significantly stronger after boxing with non-aggressive (B,C) or aggressive (B,D) target mice.

Supplementary Figure 6: Behavioral analyses from photometry experiments in the NAcSh. There were no differences in avoidance (A) or boxing (B). Stress increased freezing in the presence of non-aggressive target mice †† p=0.01 effect of stress.
